# Supplementary material for: Aging increases vulnerability to stress-induced depression via upregulation of NADPH oxidase in mice
Source: Commun Biol. 2020 Jun 5;3:292. doi: 10.1038/s42003-020-1010-5 (PMC7275057; doi:10.1038/s42003-020-1010-5)
Supplement: Supplementary file 1 — Supplementary Information [file 42003_2020_1010_MOESM1_ESM.pdf]

## Supplementary Figures

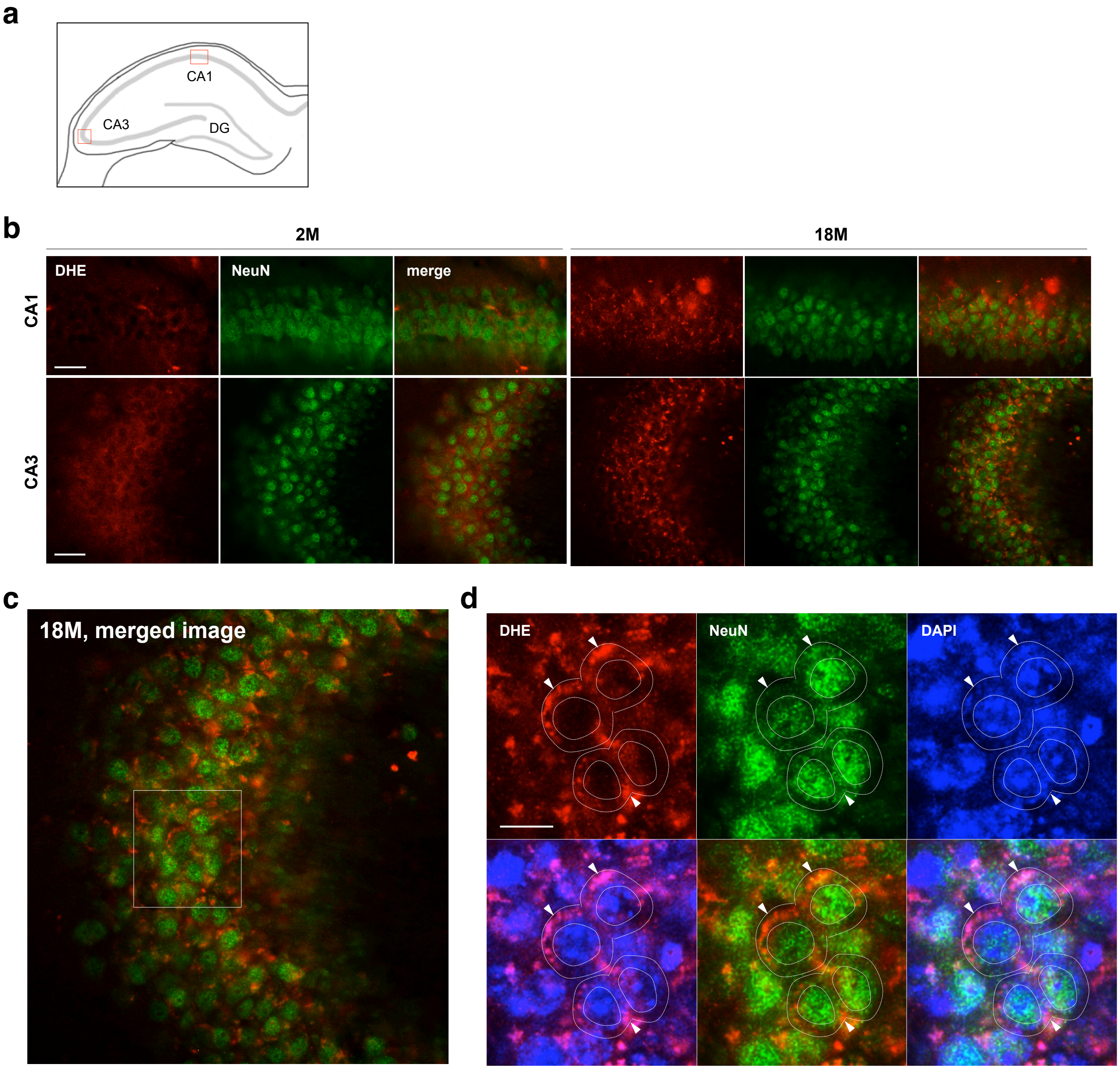


**Supplementary Fig. 1 Aged mice had increased ROS accumulation in neuronal cells of the hippocampus. a** Diagram of the hippocampus with the areas (red box) used for the high magnification images. **b** Photomicrographs showing the CA1 and CA3 regions stained with DHE and anti-NeuN in mice at 2 months (2M) and 18 months (18M) of age. Scale bars, 20 μm. **c** Enlarged merged image at the right-bottom panels of **b** (18M, CA3). **d** High magnification images showing DHE-reactive ROS and NeuN protein at the single cell level at the boxed region of **c** (white box). Circles indicate NeuN-positive cells accumulated with DHE-reactive ROS. Arrow heads, DHE-reactive ROS. DHE, red; NeuN, green. Blue, DAPI (4′,6-diamidino-2-

phenylindole)-stained nucleus. Scale bars, 20 μm.

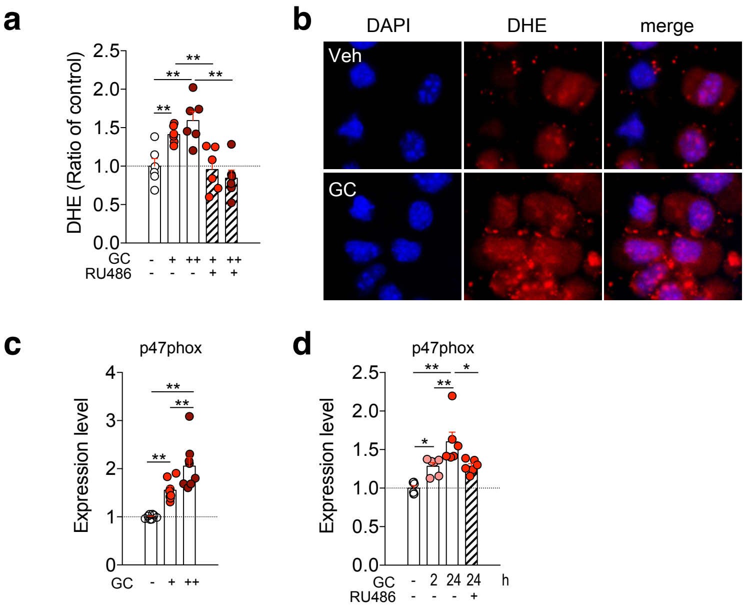


**Supplementary Fig. 2 Glucocorticoid increased ROS levels and p47phox expression in HT22 cells. a** DHE-reactive ROS levels in HT22 cells treated with glucocorticoid (GC; corticosterone) or GC plus RU486. GC (400 ng/ml) and RU486 (20 μM) were treated for 24 h (*n*=6, each; One-way ANOVA, $F(4,25)=10.49$, $p<0.0001$). **b** Visualization of DHE-reactive ROS accumulation in HT22 cells treated with GC (400 ng/ml) for 24 h. Veh, vehicle. **c** p47phox transcript levels in HT22 cells treated with GC (400, 800 ng/ml) (*n*=8, each; One-way ANOVA, $F(2,21)=23.2$, $p<0.0001$). **d** p47phox transcript levels in HT22 cells treated with GC (400 ng/ml) for 2 or 24-h, or with GC plus RU486 (20 μM) for 24 h (*n*=6, each; One-way ANOVA, $F(3,20)=12.15$, $p<0.0001$). *$p<0.05$ and **$p<0.01$. One-way ANOVA followed by Newman-Keuls post hoc test.

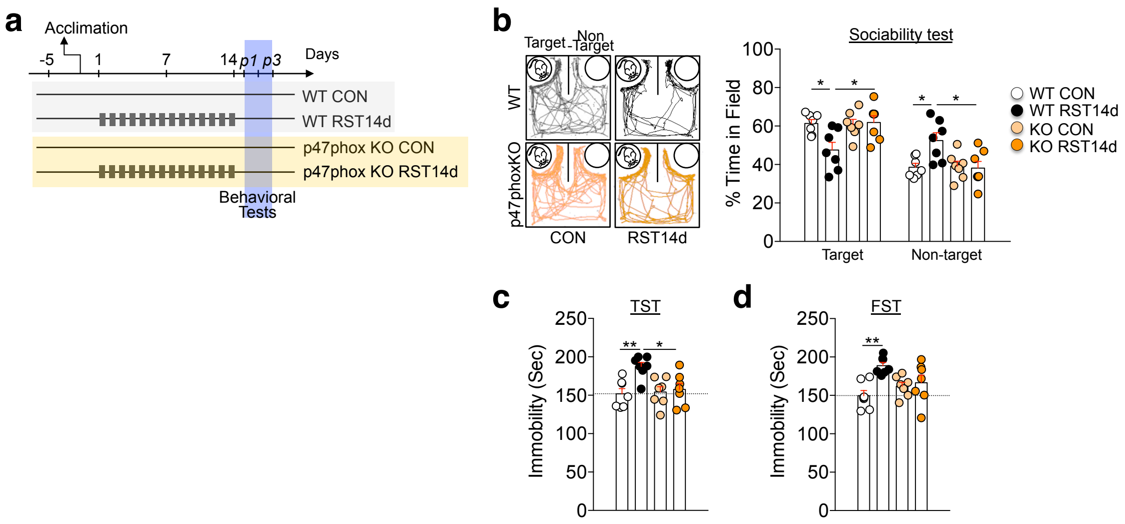


**Supplementary Fig. 3 p47phox KO mice were resilient to stress-induced depression. a** Experimental design for treatment with daily 2-h restraint for 14 days (RST14d) in wild-type and p47phox KO mice, and following behavioral tests. **b** Representative tracking plots and time spent in the target and non-target fields in the sociability test (*n*=7 mice/group; target field, Two-way ANOVA, stress, $F(1,24)=4.925$, $p=0.0362$; genotype, $F(1,24)=4.245$, $p=0.0504$; stress x genotype, $F(1,24)=5.672$, $p=0.0255$; non-target field, Two-way ANOVA, stress, $F(1,24)=4.963$, $p=0.0355$; genotype, $F(1,24)=4.304$, $p=0.0489$; stress x genotype, $F(1,24)=5.682$, $p=0.0254$). **c,d** Immobility time in the TST and FST (*n*=7 mice/group; TST, Two-way ANOVA, stress, $F(1,24)=4.039$, $p=0.0558$; genotype, $F(1,24)=8.071$, $p=0.009$; stress x genotype, $F(1,24)=5.523$, $p=0.0273$; FST, Two-way ANOVA, stress, $F(1,24)=0.5345$, $p=0.4718$, genotype, $F(1,24)=10.28$, $p=0.0038$; stress x genotype, $F(1,24)=6.411$, $p=0.0183$). $^*p<0.05$ and $^{**}p<0.01$. Two-way ANOVA followed by Bonferroni post hoc test.

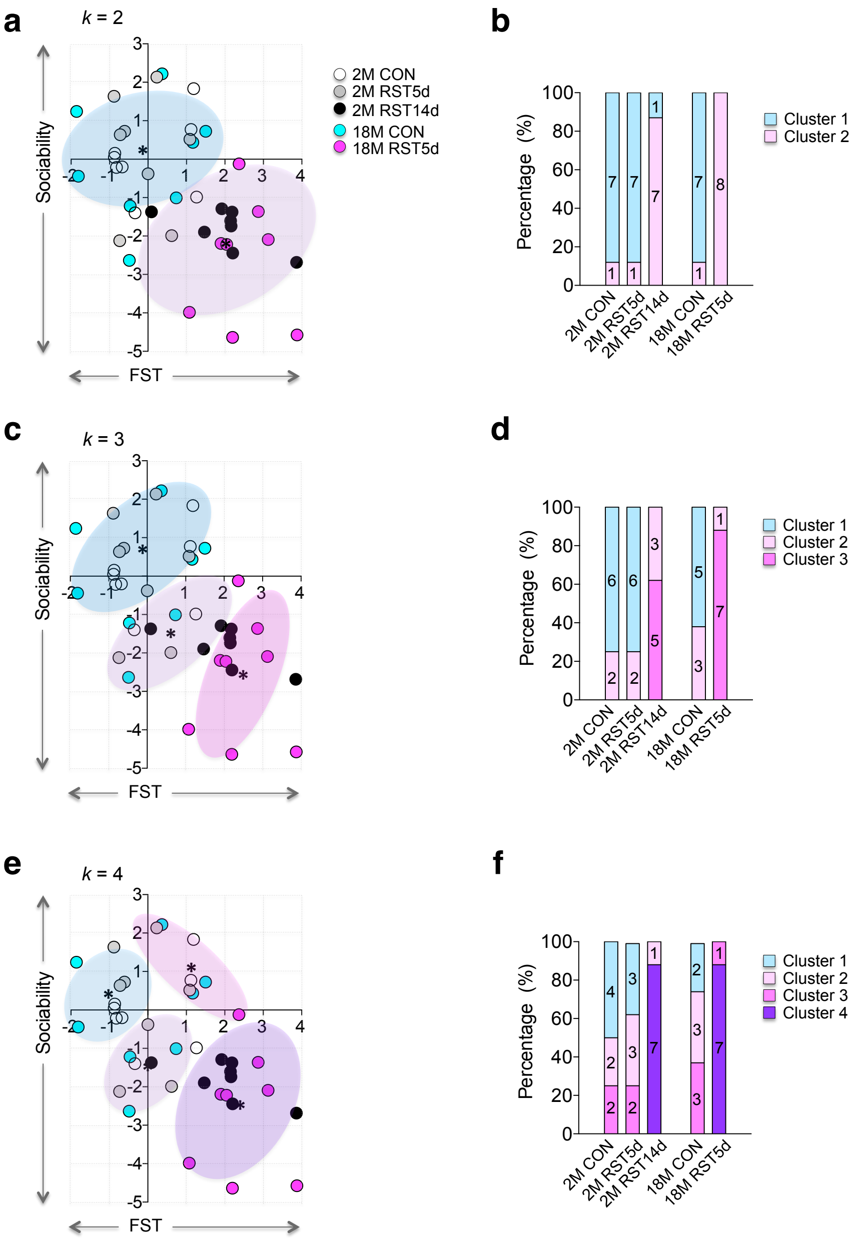


**Supplementary Fig. 4 *K*-Means cluster analyses of individuals in the sociability test x FST matrix. a** *K*-Means clustering (*k* = 2) of all individuals in the sociability test X FST matrix plotting with *z*-scores. **b** Percent proportion of each group in the two clusters (cluster 1, 87.5% for 2M CON, 87.5% for 2M RST5d, 12.5% for 2M RST14d, 87.5% for 18M CON, and 0% for 18M RST5d; cluster 2, 12.5% for 2M CON, 12.5% for 2M RST5d, 87.5% for 2M RST14d, 12.5% for 18M CON, and 100% for 18M RST5d). These data are the same as Fig. 2g,h, but presented for comparisons with the following *k* = 3 and *k* = 4 data groups. n, number of animals. **c,d** *K*-Means clustering (*k* = 3) of all

individuals in the sociability test X FST matrix and % proportion of each group in the three clusters (cluster 1, 75% for 2M CON, 75% for 2M RST5d, 0% for 2M RST14d, 62.5% for 18M CON, and 0% for 18M RST5d; cluster 2, 25% for 2M CON, 25% for 2M RST5d, 37.5% for 2M RST14d, 37.5% for 18M CON, and 12.5% for 18M RST5d; cluster 3, 0% for 2M CON, 0% for 2M RST5d, 62.5% for 2M RST14d, 0% for 18M CON, and 87.5% for 18M RST5d). **e,f** *K*-Means clustering (*k* = 4) of all individuals in the sociability test X FST matrix and % proportion of each group in the four clusters (cluster 1, 50% for 2M CON, 37.5% for 2M RST5d, 0% for 2M RST14d, 25% for 18M CON, and 0% for 18M RST5d; cluster 2, 25% for 2M CON, 37.5% for 2M RST5d, 12.5% for 2M RST14d, 37.5% for 18M CON, and 0% for 18M RST5d; cluster 3, 25% for 2M CON, 25% for 2M RST5d, 0% for 2M RST14d, 37.5% for 18M CON, and 12.5% for 18M RST5d; cluster 4, 0% for 2M CON, 0% for 2M RST5d, 87.5% for 2M RST14d, 0% for 18M CON, and 87.5% for 18M RST5d).

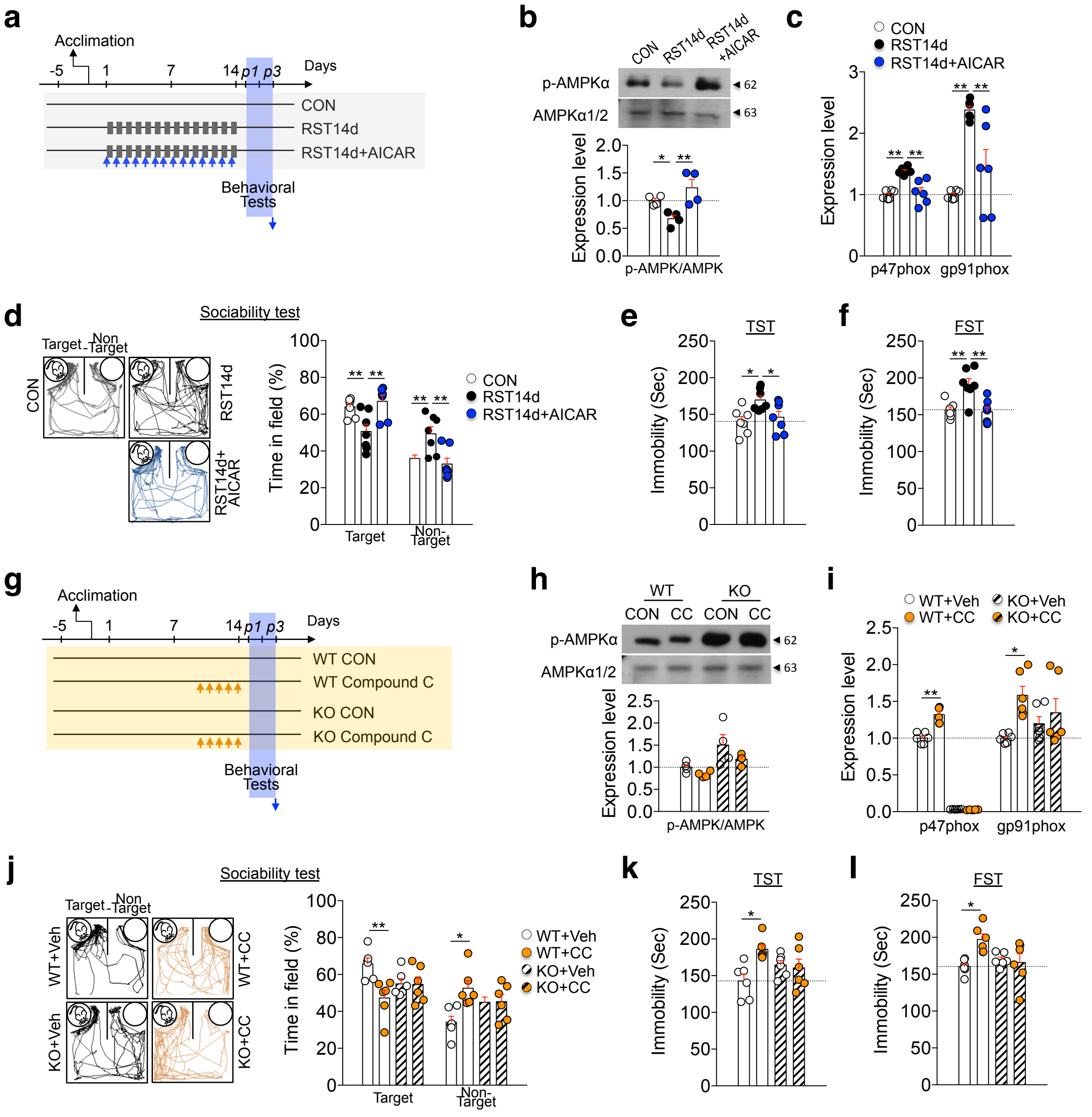


**Supplementary Fig. 5 AMPK activity was critical for p47phox and gp91phox expression and stress-induced depression. a** Experimental design for treatment with RST14d or RST14d plus AICAR (RST14d + AICAR), and following behavioral tests. AICAR; 500 mg/Kg (i.p.). Arrow, tissue preparation point. **b** Western blot data showing p-AMPK levels (*n*=4, each; One-way ANOVA, F(2,9)=8,188, *p*=0.0094). **c** p47phox and gp91phox transcript levels in the hippocampus of mice treated with RST14d or RST14d + AICAR, and their control (*n*=6, each; p47phox, One-way ANOVA, F(2,15)=18.01, *p*=0.0001; gp91phox, One-way ANOVA, F(2,15)=15.72, *p*=0.0002). **d** Representative tracking plots and time spent in the target and non-target fields in the sociability test (*n*=7 mice/group; target field, One-way

ANOVA, $F(2,18)=7.8$, $p=0.0036$; non-target field, One-way ANOVA, $F(2,18)=7.872$, $p=0.0035$). **e,f** Immobility time in the TST of mice treated with RST14d or RST14d + AICAR, and their control ($n=7$ mice/group; TST, One-way ANOVA, $F(2,18)=5.249$, $p=0.016$; FST, One-way ANOVA, $F(2,18)=11.15$, $p=0.0007$). **g** Experimental design for treatment with Compound C (CC) for 5 days in wild-type (WT) and p47phox KO mice, and following behavioral tests. CC; 10 mg/kg (i.p.), Veh, vehicle. Arrow, tissue preparation point. **h** Western blot data showing p-AMPK levels ($n=4$, each; Two-way ANOVA, genotype, $F(1,12)=3.52$, $p=0.0852$; treatment, $F(1,12)=10.87$, $p=0.0064$; genotype x treatment, $F(1,12)=0.3726$, $p=0.553$). **i** p47phox and gp91phox transcript levels in the hippocampus of WT+Veh, WT+CC, KO+Veh, and KO+CC mouse groups ($n=6$, each; p47phox, Two-way ANOVA, genotype, $F(1,20)=35.31$, $p<0.0001$; treatment, $F(1,20)=1841$, $p<0.0001$; genotype x treatment, $F(1,20)=38.57$, $p<0.0001$; gp91phox, Two-way ANOVA, genotype, $F(1,20)=8.57$, $p=0.0083$; treatment, $F(1,20)=0.02719$, $p=0.8707$; genotype x treatment, $F(1,20)=3.057$, $p=0.0957$). **j** Representative tracking plots and time spent in the target and non-target fields in the sociability test ($n=6$ mice/group; target field, Two-way ANOVA, genotype, $F(1,20)=6.782$, $p=0.017$; treatment, $F(1,20)=0.226$, $p=0.6397$; genotype x treatment, $F(1,20)=6.419$, $p=0.0198$; non-target field, Two-way ANOVA, genotype, $F(1,20)=6.787$, $p=0.0169$; treatment, $F(1,20)=0.2352$, $p=0.6329$; genotype x treatment, $F(1,20)=6.321$, $p=0.0206$). **k**,**l** Immobility time in the TST and FST of WT+Veh, WT+CC, KO+Veh, and KO+CC mouse groups ($n=6$ mice/group; TST, Two-way ANOVA, genotype, $F(1,20)=4.923$, $p=0.0382$; treatment, $F(1,20)=0.04004$, $p=0.8434$, genotype x treatment, $F(1,20)=7.102$, $p=0.0149$; FST, Two-way ANOVA, genotype, $F(1,20)=4.68$, $p=0.0428$; treatment, $F(1,20)=2.11$, $p=0.1619$, genotype x treatment, $F(1,20)=7.078$, $p=0.015$). $^{*}p<0.05$ and $^{**}p<0.01$. One-way ANOVA followed by Newman-Keuls post hoc test and two-way ANOVA followed by Bonferroni post hoc test.

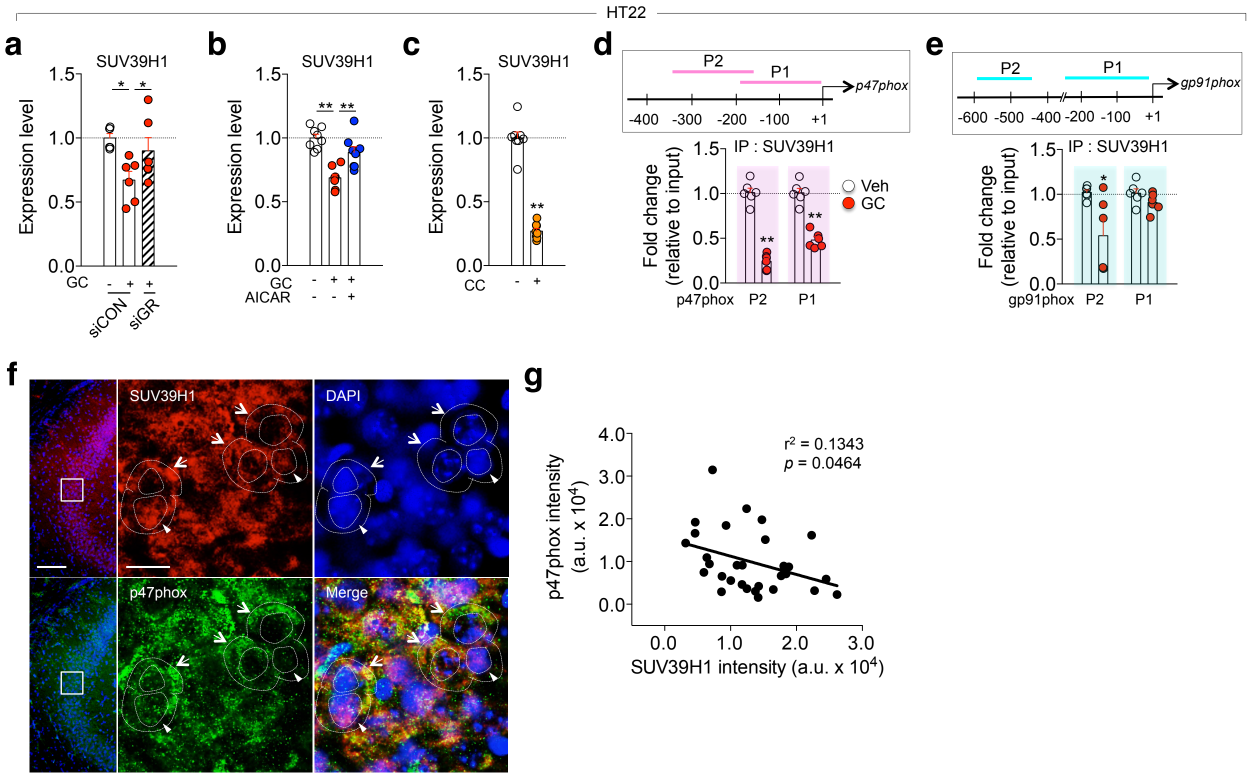


**Supplementary Fig. 6 SUV39H1 mediated GC-induced upregulation of p47phox and gp91phox expression in HT22 cells. a-c** SUV39H1 transcript levels in HT22 cells treated with GC and GC plus siGR (**a**), GC and GC plus AICAR (**b**), and CC (**c**) (**a** *n*=6, each; One-way ANOVA, $F(2,15)=5.197$, $p=0.0193$; **b** *n*=8, each; One-way ANOVA, $F(2,21)=20.38$, $p<0.0001$; **c** *n*=8, each). **d,e** ChIP-qPCR analysis showing SUV39H1 binding to the promoter of p47phox and gp91phox in HT22 cells treated with GC (*n*=6, each). **f,g** Photomicrographs showing co-localization of SUV39H1 and p47phox in CA3 pyramidal neurons of young mice, and their normalized staining intensity values. Note the inverse relationship (arrows and arrow heads) in the expression levels between SUV39H1 and p47phox (*n*=30 cells). Scale bars, 100 μm.

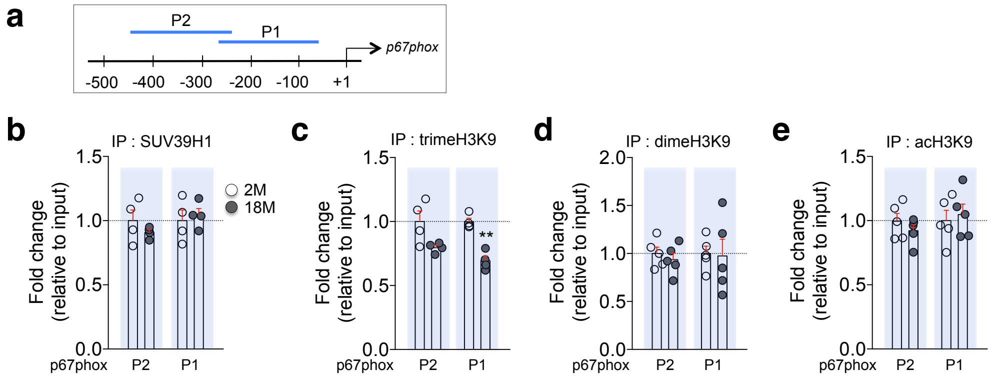


**Supplementary Fig. 7 SUV39H1 did not affect p67phox expression. a** Diagram showing the proximal promoter region of the p67phox gene. The promoter regions (P1 and P2) used for ChIP-qPCR analysis are indicated. P2, -445 ~ -231 bp; P1, -250 ~ -40 bp. **b-e** ChIP-qPCR analysis showing the levels of SUV39H1, trimeH3K9, dimeH3K9, or acH3K9 binding to the promoter of the *p67phox* in the hippocampus of mice at 2 and 18 months of age (*n*=4 for SUV39H1 and TrimeH3K9, *n*=5 for DimeH3K9 and acH3K9). **p*<0.05 and ***p*<0.01.

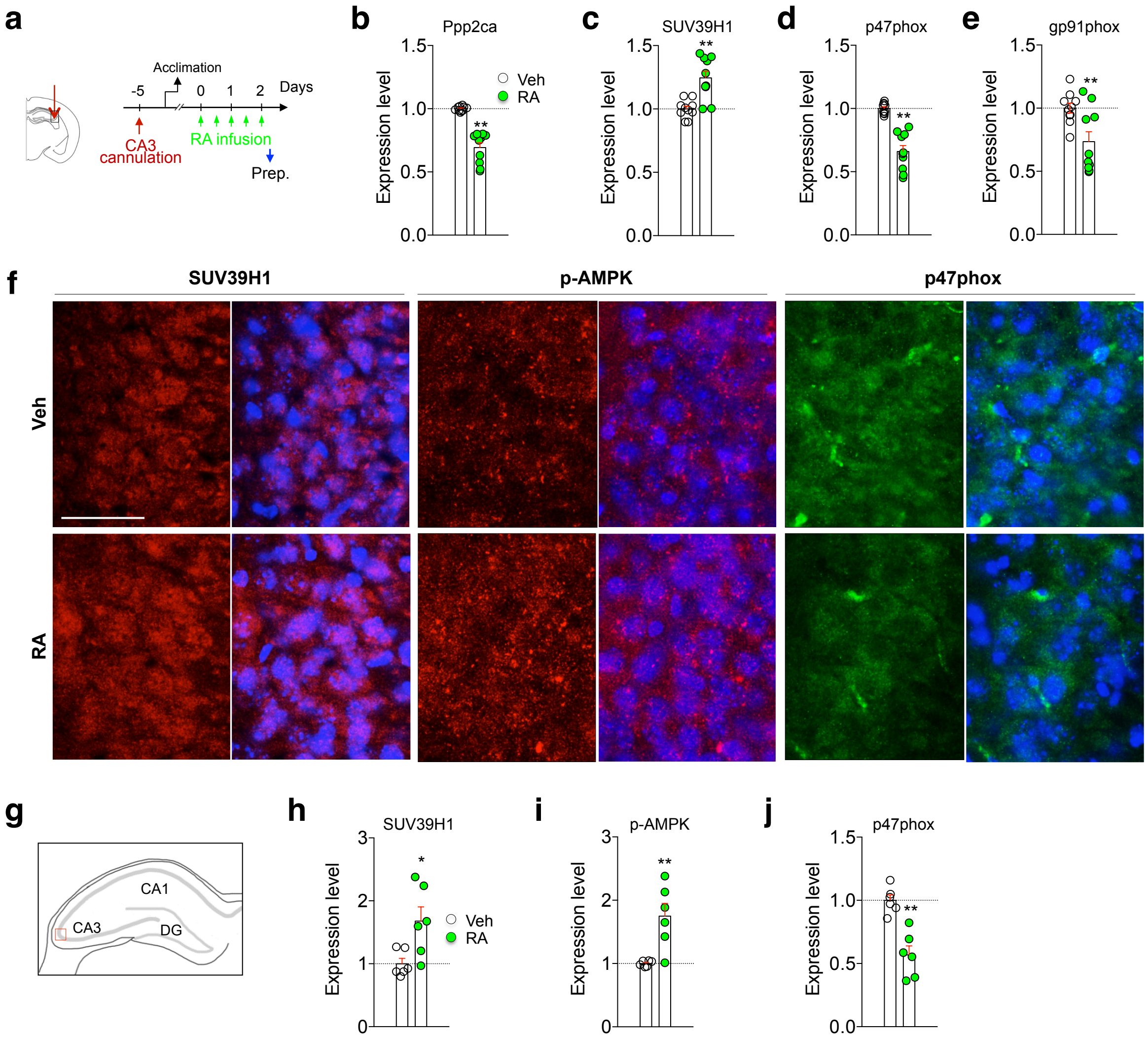


**Supplementary Fig. 8 RA reversed the changes in the Ppp2ca-p-AMPK-SUV39H1-CREB-p47phox pathway in opposition to aging and stress. a** Experimental design for direct infusion of RA into the CA3 region. **b-e** Ppp2ca, SUV39H1, p47phox, and gp91phox transcript levels in the CA3 (*n*=10, each). **f-j** Photomicrographs showing SUV39H1, p-AMPK and p47phox in CA3 pyramidal neurons of mice infused with Veh or RA and quantification (*n*=6 mice, each).

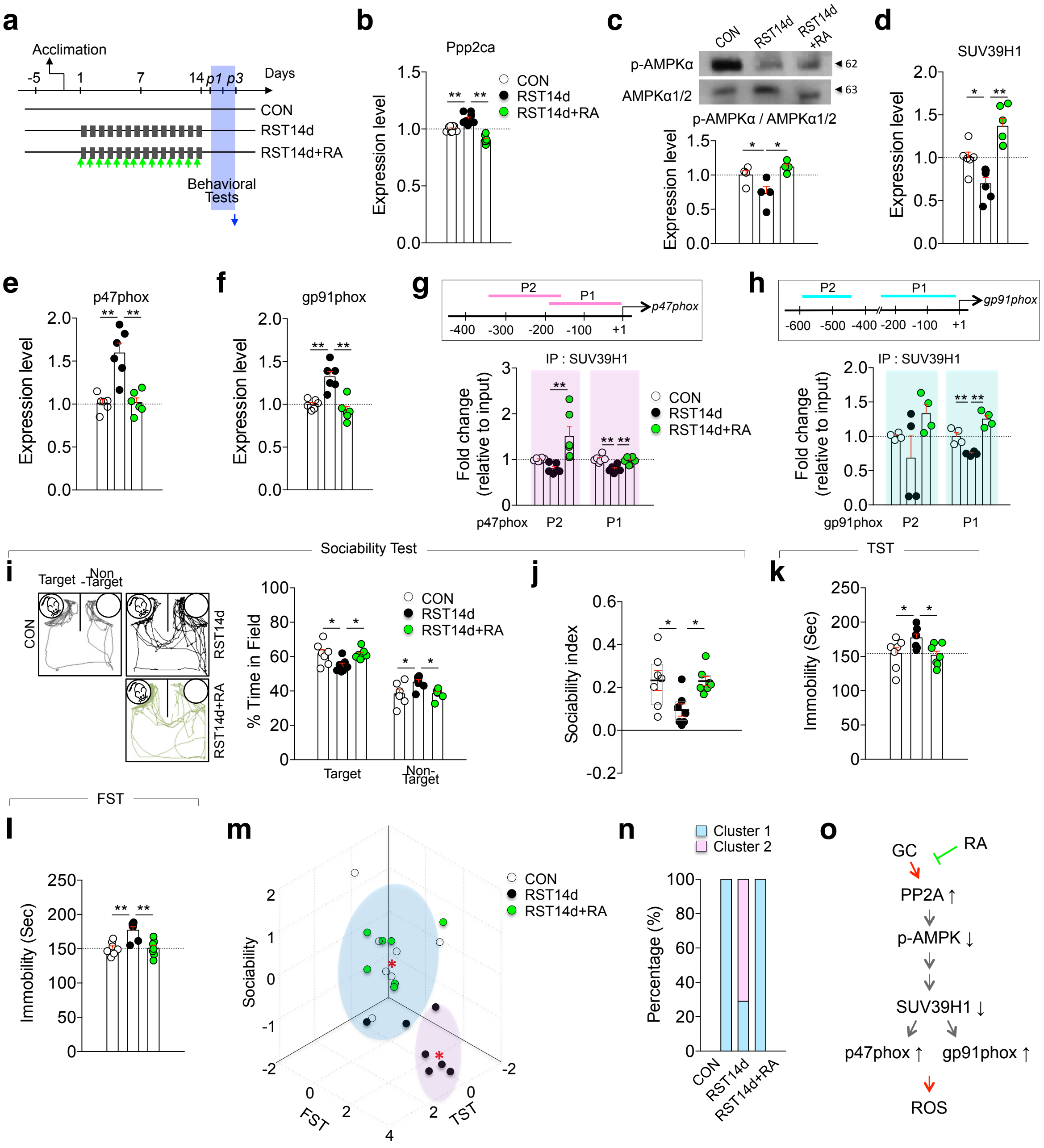

**Supplementary Fig. 9 RA blocked stress-induced changes in the Ppp2ca-SUV39H1-p47phox pathway and stress-induced depression-like behavior in young mice. a** Experimental design for treatment with daily 2-h restraint for 14 days (RST14d) or RST14d plus RA in young mice (2 M), and following behavioral tests. Arrow, time point for tissue preparation. **b** Real-time PCR data showing Ppp2ca transcript level in the hippocampus of mice treated with RAT14d or RST14d +RA (*n*=8, each; One-way ANOVA, F(2,21)=46.42, *p*<0.0001). **c** Western blot data showing the p-AMPK levels in the hippocampus of mice treated with RST14d or RST14d +RA (*n*=4, each;

One-way ANOVA, $F(2,9)=6.828$, $p=0.0157$). **d-f** Real-time PCR data showing SUV39H1, p47phox and gp91phox transcript levels in the hippocampus of mice treated with RST14d or RST14d +RA ($n=6$, each; SUV39H1, One-way ANOVA, $F(2,15)=18.76$, $p<0.0001$; p47phox, One-way ANOVA, $F(2,15)=19.29$, $p<0.0001$; gp91phox, One-way ANOVA, $F(2,15)=18.77$, $p<0.0001$). **g,h** Diagram showing the promoter region of the *p47phox* and *gp91phox*. ChIP-qPCR assay data showing the levels of SUV39H1 binding to the promoter of the *p47phox* and *gp91phox* in the hippocampus of mice treated with RST14d or RST14d +RA ($n=6$, each, p47phox P2, One-way ANOVA, $F(2,15)=8.157$, $p=0.004$; p47phox P1, One-way ANOVA, $F(2,15)=13.93$, $p=0.0004$; $n=4$, each, gp91phox P2, One-way ANOVA, $F(2,9)=2.552$, $p=0.1325$; gp91phox P1, One-way ANOVA, $F(2,9)=27.33$, $p=0.0002$). **i** Representative tracking plots and time spent in the target and non-target fields ($n=7$ mice/group; target field, One-way ANOVA, $F(2,18)=5.334$, $p=0.0152$; non-target field, One-way ANOVA, $F(2,18)=5.252$, $p=0.016$). **j** Sociability index in the sociability test ($n=7$ mice/group; One-way ANOVA, $F(2,18)=5.291$, $p=0.0156$). **k,l** Immobility time in the TST and FST ($n=7$ mice/group; TST, One-way ANOVA, $F(2,18)=4.203$, $p=0.0318$; FST, One-way ANOVA, $F(2,18)=12.05$, $p=0.0005$). **m,n** *K*-Means clustering of individuals in the sociability x TST x FST matrix plotting with *z*-scores and % composition of each group in the clusters (cluster1, 100% for CON, 28.6% for RST14d, and 100% for RST14d+RA; cluster2, 0% for CON, 71.4% for RST14d, and 0% for RST14d+RA). **o** A summary of the signaling pathway of PP2A, p-AMPK, SUV39H1, p47phox, and gp91phox in aging and stress-induced depression, and putative RA action site. *$p<0.05$ and **$p<0.01$. One-way ANOVA followed by Newman-Keuls post hoc test.
