# Supplementary material for: Aging increases vulnerability to stress-induced depression via upregulation of NADPH oxidase in mice
Source: Commun Biol. 2020 Jun 5;3:292. doi: 10.1038/s42003-020-1010-5 (PMC7275057; doi:10.1038/s42003-020-1010-5)

Supplementary Data1 - WB blot images

Shown in Fig. 3c

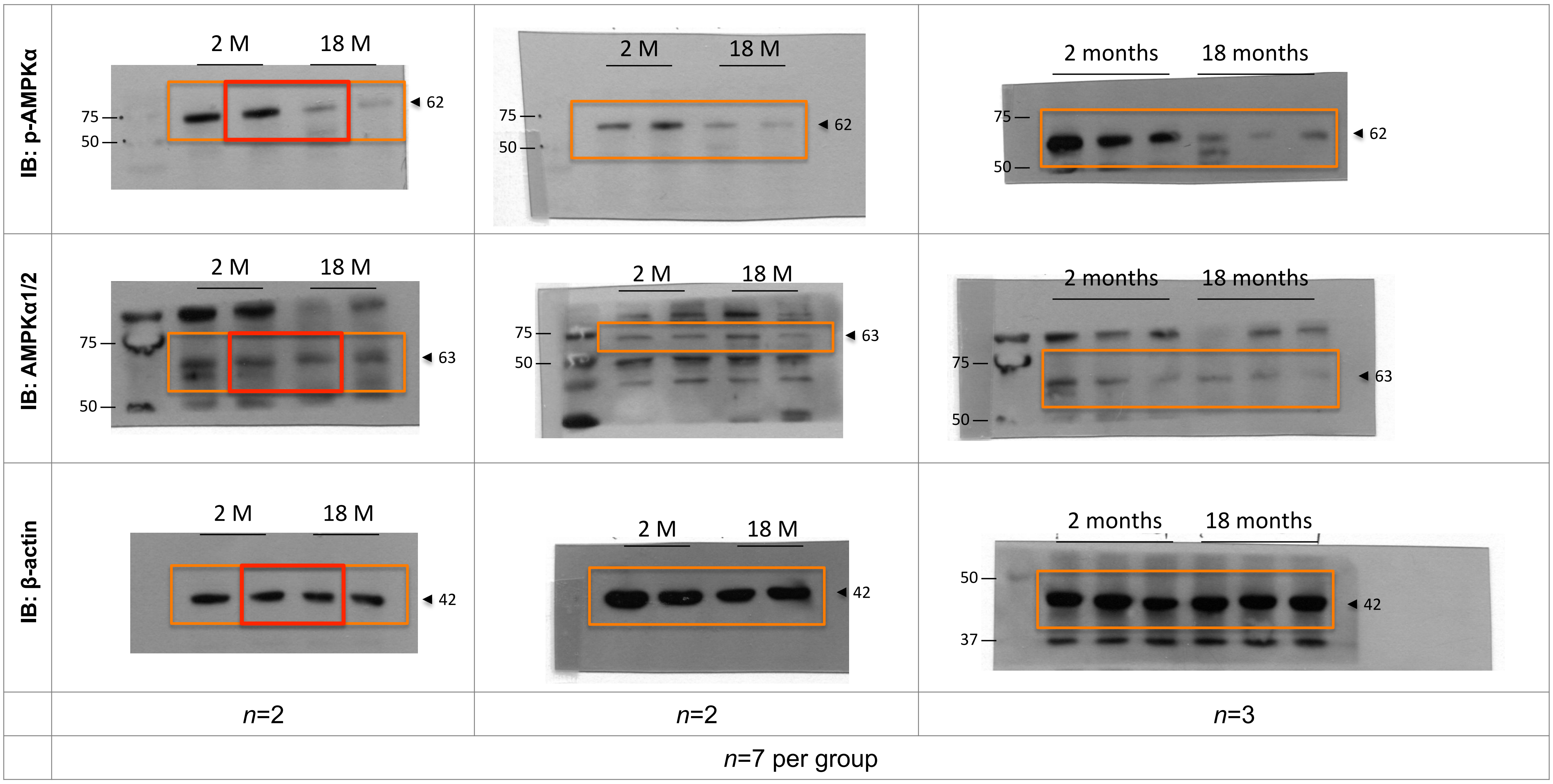

Shown in Fig. 3f

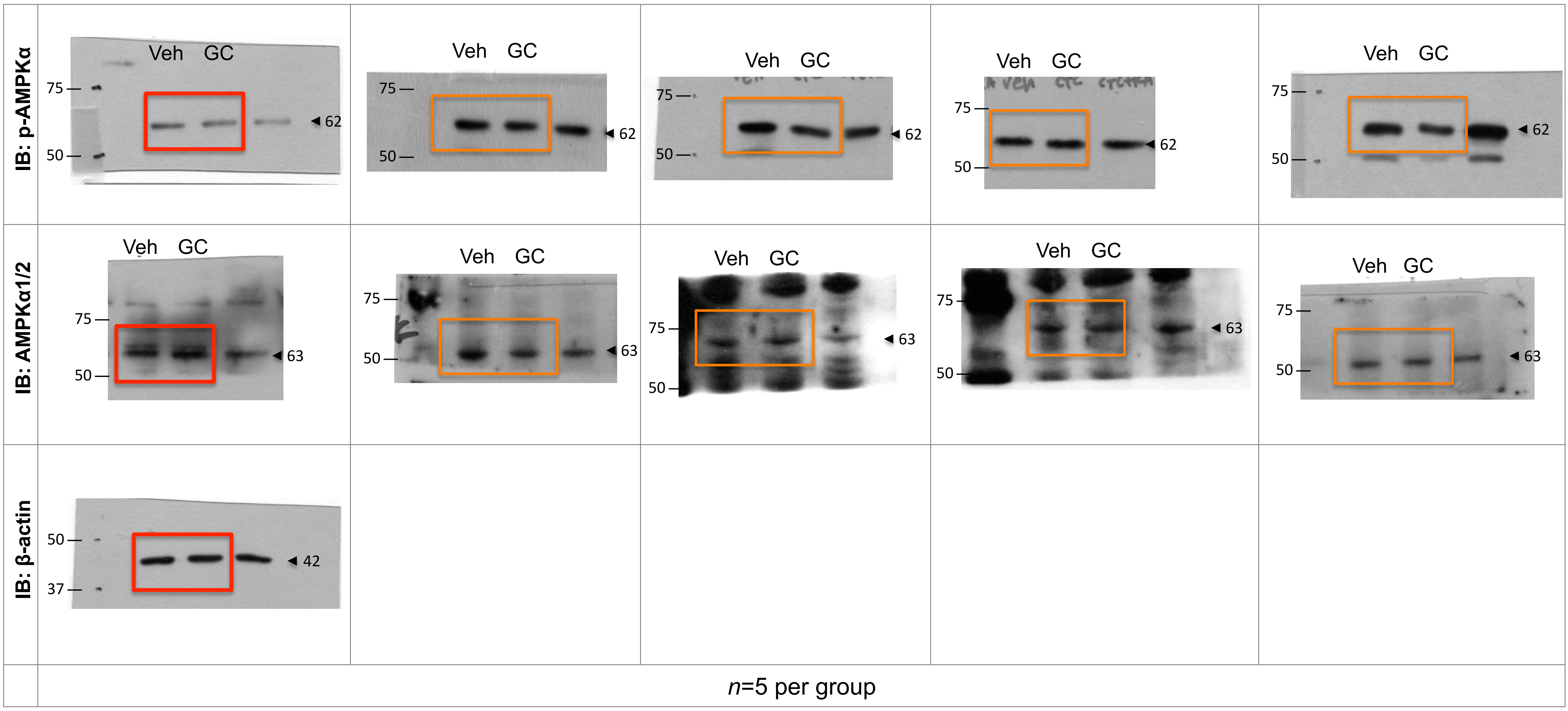

Shown in Fig. 4d

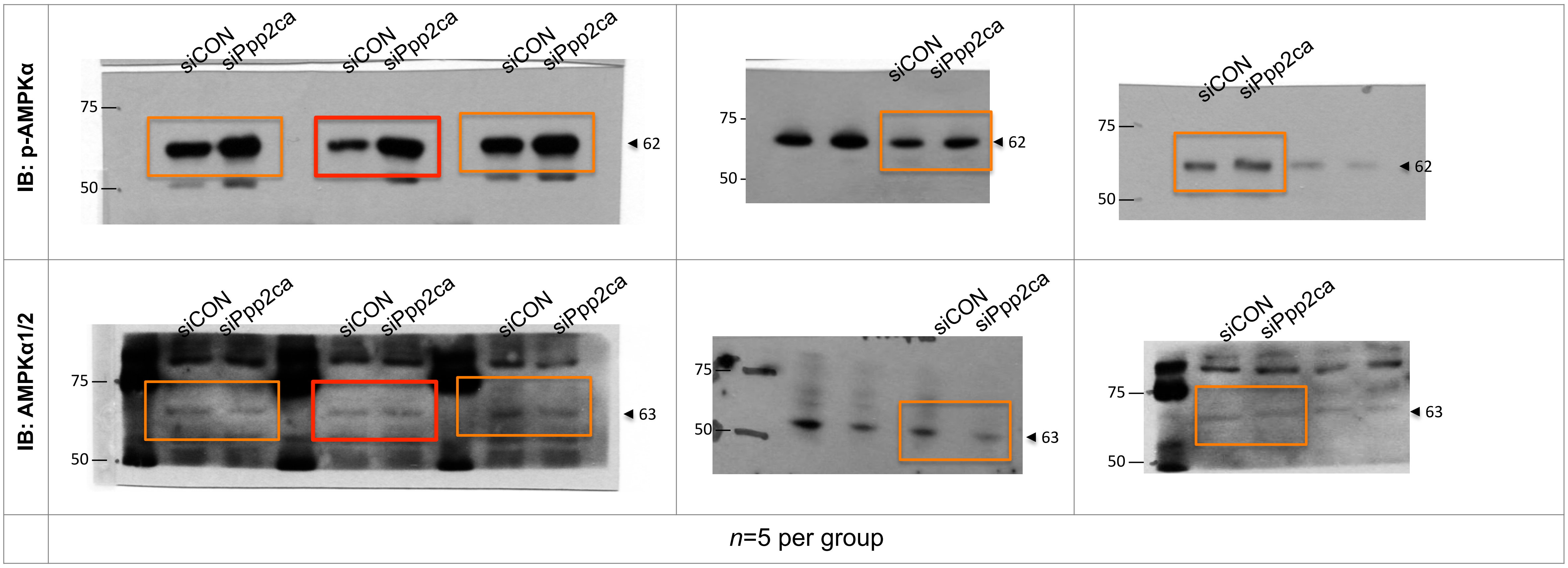

Shown in Fig. 5l

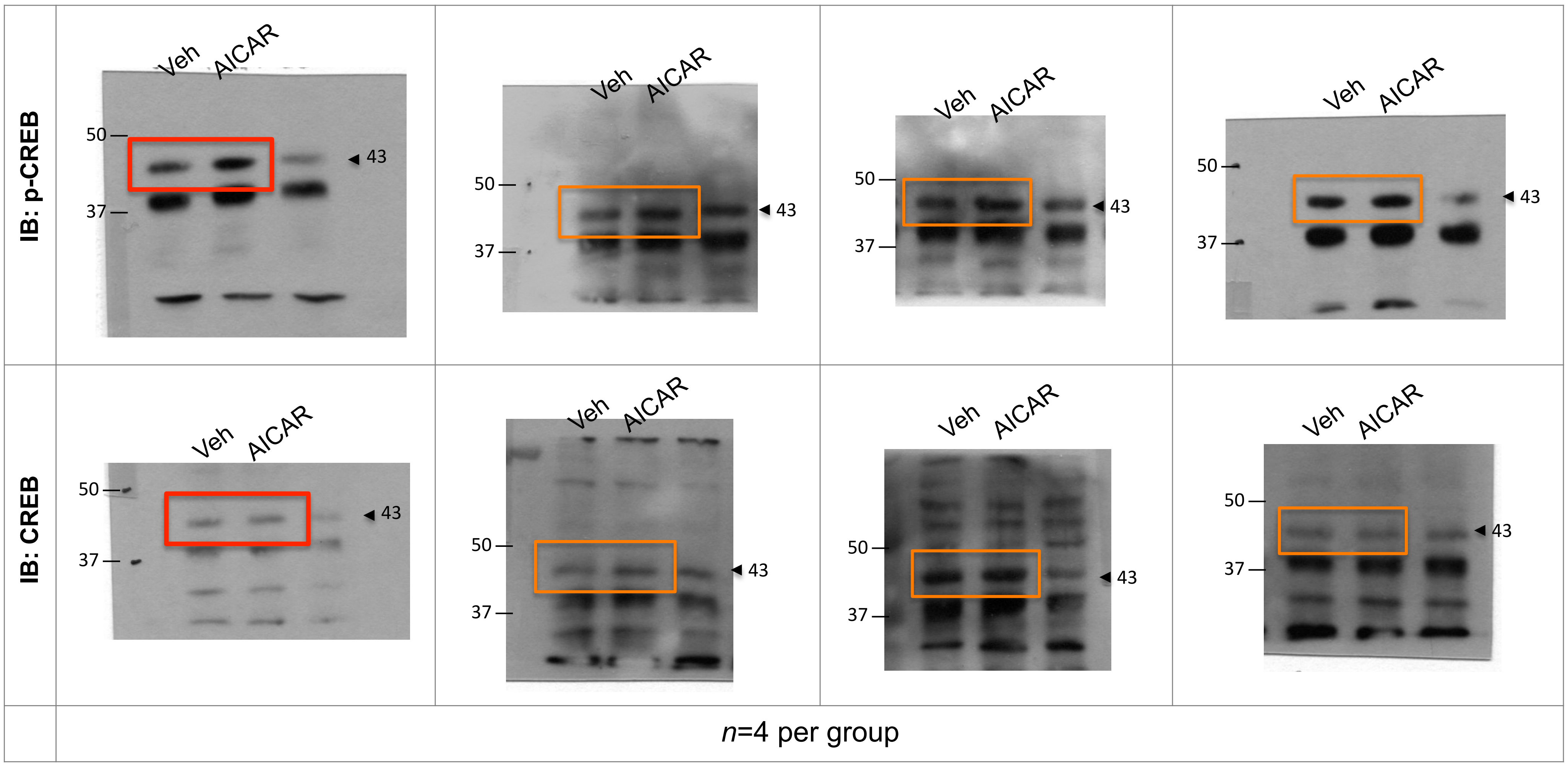

Shown in Fig. 5m

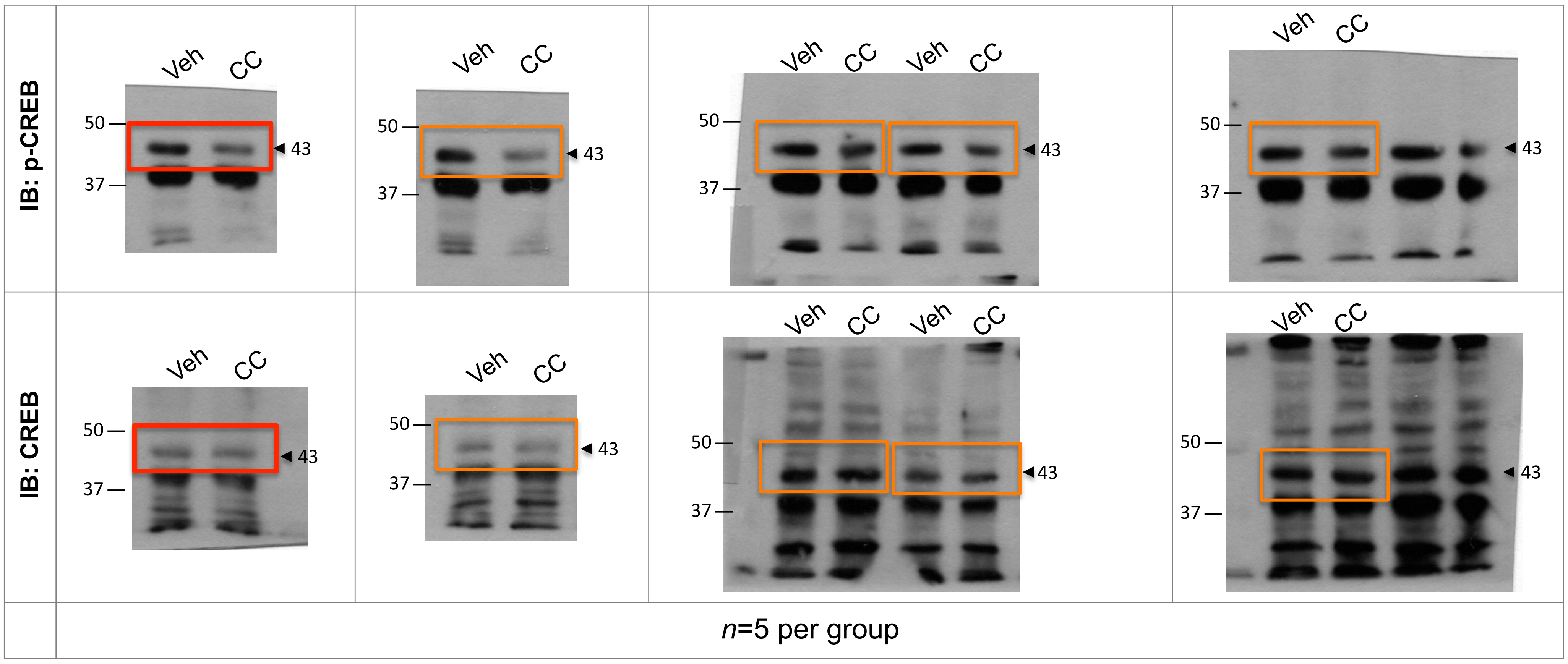

Shown in Supplemental Fig. 5b

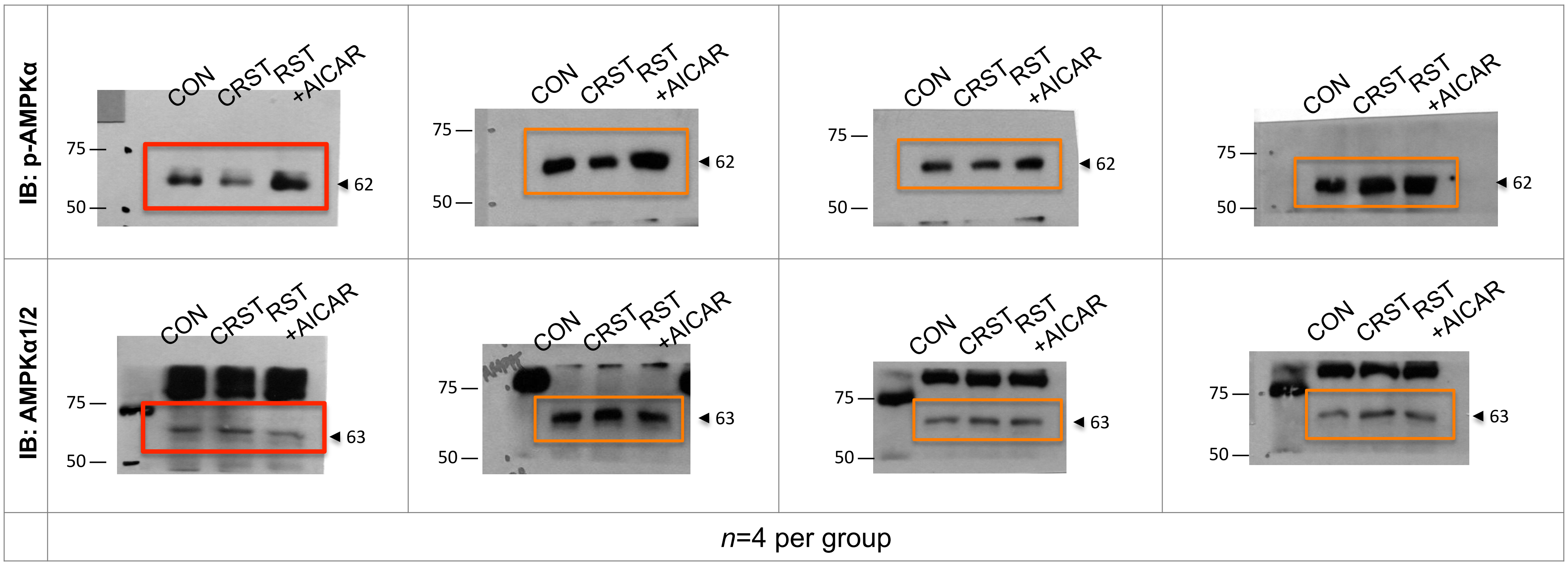

Shown in Supplemental Fig. 5h

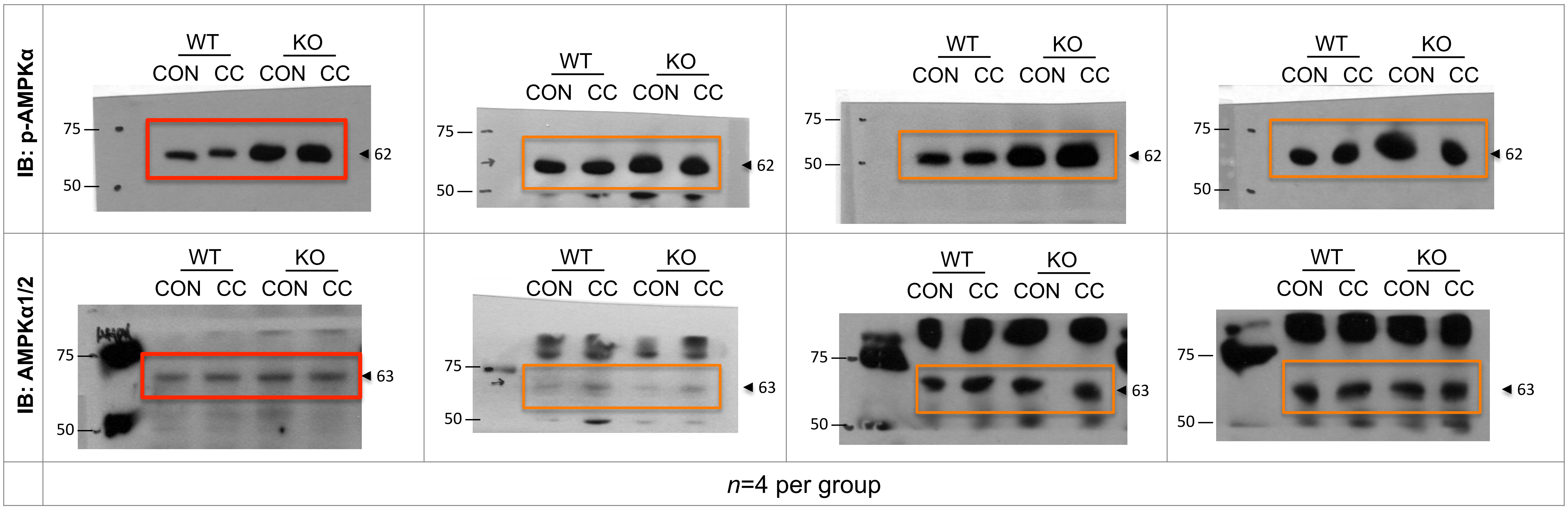

Shown in Supplemental Fig. 9c

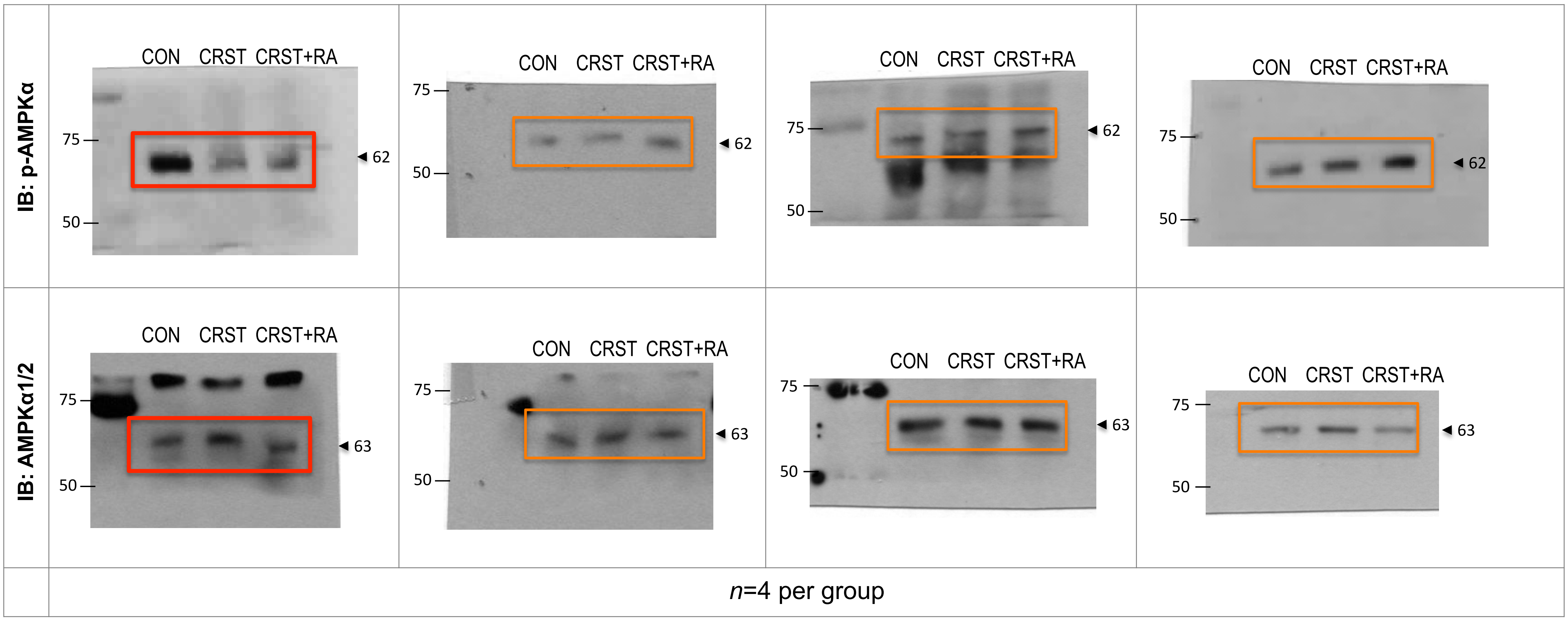

Supplement: Supplementary file 2 — Supplementary Data 1 [file 42003_2020_1010_MOESM2_ESM.pdf]
