# Supplementary material for: Aging increases vulnerability to stress-induced depression via upregulation of NADPH oxidase in mice
Source: Commun Biol. 2020 Jun 5;3:292. doi: 10.1038/s42003-020-1010-5 (PMC7275057; doi:10.1038/s42003-020-1010-5)
Supplement: Supplementary file 5 — Reporting Summary [file 42003_2020_1010_MOESM5_ESM.pdf]

nature research

Corresponding author(s): Pyung-Lim Han

Last updated by author(s): 04/27/2020

Reporting Summary

Nature Research wishes to improve the reproducibility of the work that we publish. This form provides structure for consistency and transparency in reporting. For further information on Nature Research policies, see Authors & Referees and the Editorial Policy Checklist .

Statistics

For all statistical analyses, confirm that the following items are present in the figure legend, table legend, main text, or Methods section.

n/a	Confirmed	
☐	☒	The exact sample size (*n*) for each experimental group/condition, given as a discrete number and unit of measurement
☐	☒	A statement on whether measurements were taken from distinct samples or whether the same sample was measured repeatedly
☐	☒	The statistical test(s) used AND whether they are one- or two-sided<br>*Only common tests should be described solely by name; describe more complex techniques in the Methods section.*
☒	☐	A description of all covariates tested
☒	☐	A description of any assumptions or corrections, such as tests of normality and adjustment for multiple comparisons
☐	☒	A full description of the statistical parameters including central tendency (e.g. means) or other basic estimates (e.g. regression coefficient) AND variation (e.g. standard deviation) or associated estimates of uncertainty (e.g. confidence intervals)
☒	☐	For null hypothesis testing, the test statistic (e.g. *F*, *t*, *r*) with confidence intervals, effect sizes, degrees of freedom and *P* value noted<br>*Give P values as exact values whenever suitable.*
☒	☐	For Bayesian analysis, information on the choice of priors and Markov chain Monte Carlo settings
☒	☐	For hierarchical and complex designs, identification of the appropriate level for tests and full reporting of outcomes
☒	☐	Estimates of effect sizes (e.g. Cohen's *d*, Pearson's *r*), indicating how they were calculated

Our web collection on statistics for biologists contains articles on many of the points above.

Software and code

Policy information about availability of computer code

Data collection	CFX Manager Software ver. 3.1, SoftMax Pro software ver. 5.4, video tracking system SMART, Image-Pro Premier 6.0, myassays.com (for Enzo Life Sciences EIA kit), and MetaMorph Microscopy Automation & Image Analysis software.
Data analysis	SPSS Statistics 25 software and GraphPad PRISM 6.0 software.

For manuscripts utilizing custom algorithms or software that are central to the research but not yet described in published literature, software must be made available to editors/reviewers. We strongly encourage code deposition in a community repository (e.g. GitHub). See the Nature Research guidelines for submitting code & software for further information.

Data

Policy information about availability of data

All manuscripts must include a data availability statement. This statement should provide the following information, where applicable:

- Accession codes, unique identifiers, or web links for publicly available datasets
- A list of figures that have associated raw data
- A description of any restrictions on data availability

All data generated or analysed during this study are included in this article and its Supplementary information files.

Field-specific reporting

Please select the one below that is the best fit for your research. If you are not sure, read the appropriate sections before making your selection.

☒ Life sciences ☐ Behavioural & social sciences ☐ Ecological, evolutionary & environmental sciences

Life sciences study design

All studies must disclose on these points even when the disclosure is negative.

Sample size	Sample size included in its supplementary information files.
Data exclusions	Mice with mislocalized injections were excluded from the final data of stereotaxic injection experiment.
Replication	Replication of results was successful.
Randomization	applied
Blinding	applied

Reporting for specific materials, systems and methods

We require information from authors about some types of materials, experimental systems and methods used in many studies. Here, indicate whether each material, system or method listed is relevant to your study. If you are not sure if a list item applies to your research, read the appropriate section before selecting a response.

Materials & experimental systems

n/a	Involved in the study	
☐	☒	Antibodies
☐	☒	Eukaryotic cell lines
☒	☐	Palaeontology
☐	☒	Animals and other organisms
☒	☐	Human research participants
☒	☐	Clinical data

Methods

n/a	Involved in the study	
☒	☐	ChIP-seq
☒	☐	Flow cytometry
☒	☐	MRI-based neuroimaging

Antibodies

Antibodies used

1. p-AMPKα (2535s; RRID:AB_331250, Cell Signaling)
2. AMPKα1/2 (sc-74461; RRID:AB_1118940, Santa Cruz)
3. p-CREB (o6-519, RRID:AB_310153, Millipore)
4. CREB (sc-186, RRID:AB_2086021, Santa Cruz)
5. β-actin (sc-47778, RRID:AB_626632, Santa Cruz)
6. p47phox (sc-7660; RRID:AB_2298320, Santa Cruz)
7. NeuN (MAB377; RRID:AB_2298772, Millipore)
8. SUV39H1 (GTX112263; RRID:AB_1952113, GeneTex)
9. acH3K9 (ab10812; RRID:AB_297491, Abcam)
10. dimeH3K9 (ab1220; RRID:AB_449854, Abcam)
11. trimeH3K9 (ab8898; RRID:AB_306848, Abcam)
12. Dylight 594 (DI-1094; RRID:AB_2336414, DI-2594; RRID:AB_2336412)
13. fluorescein isothiocyanate (FITC; sc-2024; RRID:AB_631727, Santa Cruz)

Validation

1. validated for WB and IF.
2-5. validated for WB.
6-7. validated for IF.
8. validated for IF and ChIP.
9-11. validated for ChIP.

Eukaryotic cell lines

Policy information about cell lines

Cell line source(s)	HT22 hippocampal cells
Authentication	Seo et al. NADPH oxidase mediates depressive behavior induced by chronic stress in mice. J Neurosci. 32, 9690-9699 (2012)
Mycoplasma contamination	not tested

Commonly misidentified lines (See ICLAC register)	No commonly misidentified cell lines used

Animals and other organisms

Policy information about studies involving animals; ARRIVE guidelines recommended for reporting animal research

Laboratory animals	7-weeks male C57Bl/6 mice were purchased from a local company.<br>p47phox knockout (Stock No. 004742) and aged wild-type (C57BL/6J) mice obtained from the Jackson Laboratory.
Wild animals	Not applicable
Field-collected samples	Not applicable
Ethics oversight	All animals were handled in accordance with the animal-care guidelines of QQQ University (IACUC 16-018).

Note that full information on the approval of the study protocol must also be provided in the manuscript.
